# Neutral and charged inter-valley biexcitons in monolayer MoSe_2_

**DOI:** 10.1038/ncomms15552

**Published:** 2017-06-28

**Authors:** Kai Hao, Judith F. Specht, Philipp Nagler, Lixiang Xu, Kha Tran, Akshay Singh, Chandriker Kavir Dass, Christian Schüller, Tobias Korn, Marten Richter, Andreas Knorr, Xiaoqin Li, Galan Moody

**Affiliations:** 1 Department of Physics and Center for Complex Quantum Systems, University of Texas at Austin, Austin, Texas 78712, USA; 2 Institut für Theoretische Physik, Nichtlineare Optik und Quantenelektronik, Technische Universität Berlin, 10623 Berlin, Germany; 3 Department of Physics, University of Regensburg, 93040 Regensburg, Germany; 4 National Institute of Standards & Technology, Boulder, Colorado 80305, USA

## Abstract

In atomically thin transition metal dichalcogenides (TMDs), reduced dielectric screening of the Coulomb interaction leads to strongly correlated many-body states, including excitons and trions, that dominate the optical properties. Higher-order states, such as bound biexcitons, are possible but are difficult to identify unambiguously using linear optical spectroscopy methods. Here, we implement polarization-resolved two-dimensional coherent spectroscopy (2DCS) to unravel the complex optical response of monolayer MoSe_2_ and identify multiple higher-order correlated states. Decisive signatures of neutral and charged inter-valley biexcitons appear in cross-polarized two-dimensional spectra as distinct resonances with respective ∼20 and ∼5 meV binding energies—similar to recent calculations using variational and Monte Carlo methods. A theoretical model considering the valley-dependent optical selection rules reveals the quantum pathways that give rise to these states. Inter-valley biexcitons identified here, comprising of neutral and charged excitons from different valleys, offer new opportunities for developing ultrathin biexciton lasers and polarization-entangled photon sources.

## Abstract (web summary)

Atomically thin transition metal dichalcogenides host excitons and trions, however higher-order states, although possible, are difficult to identify experimentally. Here, the authors perform polarization-resolved coherent spectroscopy to unveil the signature of neutral and charged inter-valley biexcitons in monolayer MoSe_2_.

The formation of tightly bound excitons (electron-hole pairs)^1^,^2^,^3^,^4^,^5^ and trions (charged excitons)^6^,^7^,^8^,^9^,^10^ in atomically thin transition metal dichalcogenides (TMDs) suggests that higher-order states comprising of correlations between four or more particles are possible. A bound biexciton, resulting from four-particle correlations between two electrons and two holes, is a hallmark of many-body interactions between quasiparticles in semiconductors. Experimental identification and microscopic calculations of biexcitons in conventional semiconductors and their heterostructures have greatly advanced our fundamental understanding of many-body physics in semiconductors^11^,^12^,^13^,^14^,^15^. A biexciton is a classic example for which a mean field (Hartree–Fock) theoretical description clearly fails and higher-order electronic correlations have to be included^16^,^17^,^18^,^19^.

Investigation of biexcitons in monolayer TMDs presents new challenges compared to conventional semiconductors. Microscopic calculations of biexcitons need to properly consider non-local screening of the Coulomb interaction and the coupled spin and valley pseudospin degrees of freedom. Using left (*σ*+) or right (*σ*−) circularly polarized light tuned close to the lowest-energy *A*-exciton resonance^20^,^21^,^22^, excitons and trions are preferentially created in the *K* and *K*′ valleys at the corners of the first Brillouin zone, and the valley index can be preserved during emission processes. Questions regarding the valley degree of freedom associated with higher-order bound states naturally arise. Thus, experimental studies that can clearly identify possible bound states between excitons and trions and the associated internal valley indices are critical in guiding the development of a microscopic theory describing these higher-order correlated states in atomically thin semiconductors.

While a few experimental studies have reported the bound biexciton in monolayer TMDs, significant discrepancies between the measured^23^,^24^,^25^,^26^,^27^ and calculated^28^,^29^,^30^,^31^,^32^ binding energy remain, which may arise for several reasons. Experimentally, associating specific spectroscopic features with biexciton states can be extremely challenging. In photoluminescence (PL) spectroscopy of WSe_2_ and WS_2_ TMD monolayers, the emergence of a new peak whose intensity grows super-linearly with pump fluence has been assigned to the neutral biexciton, but resonances associated with the charged biexciton or electron plasma excitations may also appear at a similar energy^33^. Furthermore, the emission from excitons localized by impurities and defects can also increase super-linearly with pump fluence^34^,^35^. In nonlinear pump/probe experiments of monolayer MoS_2_, a negative signal at energies below the exciton resonance was attributed to evidence of induced absorption from the exciton-to-biexciton transition; however, bandgap renormalization can also give rise to a similar negative pump/probe signal^36^. Theoretically, calculations of higher-order correlated states such as biexcitons necessarily involve certain approximations, and thus, rely on the experimentally measured binding energies as guidance.

In this work, we shed new light on higher-order bound states by identifying and distinguishing neutral and charged biexcitons in monolayer MoSe_2_ using resonant two-dimensional coherent spectroscopy (2DCS)^37^. We show that a comparison of co- and cross-circularly polarized 2D spectra reveals previously unobserved resonances that are identified as neutral and charged biexciton states with binding energies of ∼20 and ∼5 meV, respectively. The neutral biexciton binding energy is significantly smaller compared to previously reported experimental values for other monolayer TMDs but agrees with more recent theoretical studies^28^,^29^,^30^,^31^,^32^. The polarization dependence of the 2D spectrum is reproduced with density matrix calculations taking into account the valley-dependent optical selection rules. These results suggest that both types of biexcitons are inter-valley in nature and consist of two excitons with large difference in crystal momentum, making them unique types of higher-order bound states with no direct analogue in conventional semiconductors.

## Results

### Linear optical response of monolayer MoSe_2_

**Figure 1:**
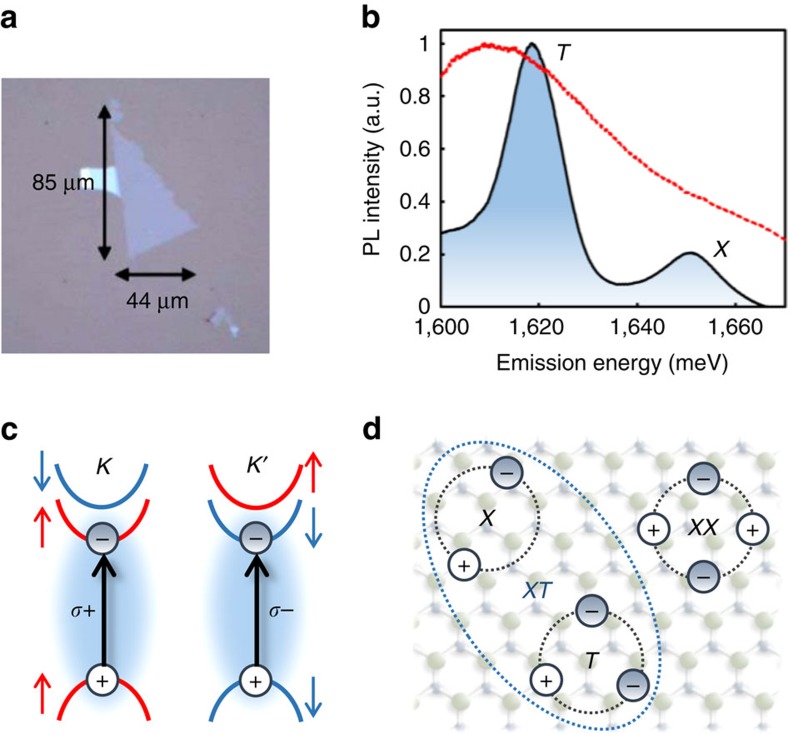
Excitons, biexcitons and trions in monolayer MoSe_2_. (**a**) Optical microscope image of the monolayer sample. (**b**) Photoluminescence spectrum taken at 13 K featuring two peaks near ∼1,650 and ∼1,620 meV attributed to the exciton (*X*) and trion (*T*), respectively. The excitation laser spectrum for the nonlinear spectroscopy experiments is indicated by the dashed red curve. (**c**) Lowest-energy direct-gap transitions at the *K* and *K*′ valleys associated with *A*-excitons are accessible using *σ*+ and *σ*− circularly polarized light, respectively. The valence band associated with the *B*-exciton is omitted for clarity. (**d**) Illustration of an exciton (*X*), trion (*T*), neutral biexciton (*XX*) and charged biexciton (*XT*) in monolayer MoSe_2_.

We examined monolayer MoSe_2_ mechanically exfoliated onto a sapphire substrate (see Fig. 1a), which is held at a temperature of 20 K for optical spectroscopy experiments in transmission. A time-integrated photoluminescence spectrum acquired using continuous-wave 532 nm excitation is shown in Fig. 1b. The spectrum features two peaks at ∼1,650 and ∼1,620 meV that have been previously assigned to the exciton and negative trion, respectively^38^. The exciton comprises an electron-hole pair in the same valley with spins illustrated in Fig. 1c. For the trion, the lowest-energy transition consists of an exciton in one valley bound to an additional electron in the opposite valley (see Supplementary Fig. 1). Biexciton resonances are not isolated in the photoluminescence spectrum due to spectral overlap with the relatively broad inhomogeneous linewidths for the exciton and trion. In principle, the lowest-energy bound biexciton transition comprises two excitons in opposite valleys, since the energy for bound biexcitons generated by simultaneous excitation of two excitons in the same valley is much higher^25^,^28^. Because the lowest-energy dipole-allowed transition in MoSe_2_ is between the highest valence band and lowest conduction band, the possible biexciton, trion and exciton configurations (see Fig. 1d) are simplified compared to tungsten-based TMDs (ref. 39).

### 2DCS of monolayer MoSe_2_

**Figure 2:**
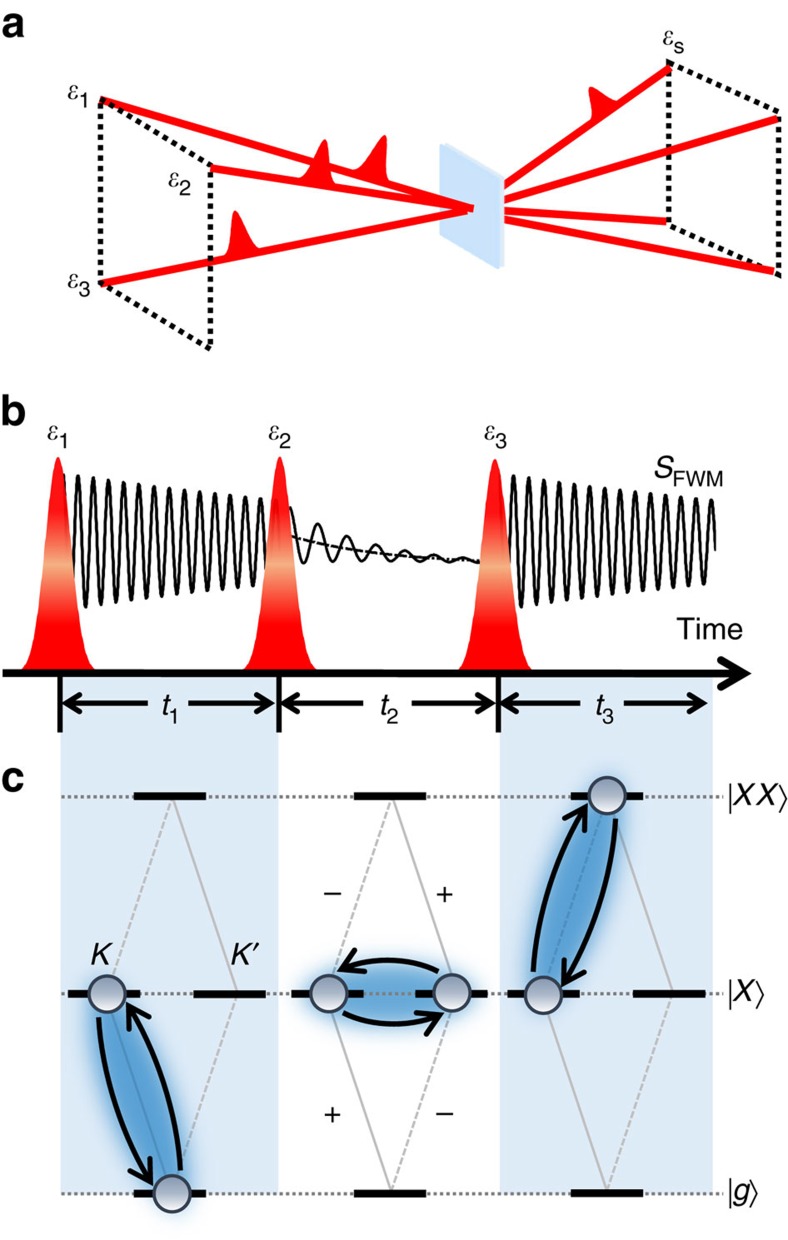
Illustration of 2DCS of biexcitons. (**a**) Box geometry used for the 2DCS experiments. Three pulses interact nonlinearly with the sample to generate a four-wave mixing signal that is collected in transmission and detected through heterodyne spectral interferometry. (**b**) Time ordering of the excitation pulses and detected signal. (**c**) The quantum pathway for accessing the bound biexciton (*XX*) using cross-circular polarization. Interactions between two excitons are modelled using a four-level system. The first pulse (*σ*+) creates an exciton coherence in the *K* valley during *t*_1_. The second pulse (*σ*−) excites the exciton transition in the *K*′ valley. The third pulse (*σ*+) drives the transition between biexciton and exciton states, which radiates as the four-wave mixing signal.

To probe biexcitons in monolayer MoSe_2_, we performed 2DCS experiments with carefully chosen excitation and polarization conditions. 2DCS is a three-pulse four-wave mixing (photon echo) technique with interferometric precision of the timing delays between the pulses and phase stabilization better than *λ*/100, where *λ* is the excitation wavelength^40^,^41^. The laser spectrum is centred near 1,620 meV (red curve in Fig. 1d), which is tuned below the exciton resonance energy to preferentially enhance any signatures of lower-energy bound biexcitons. The 2DCS experiments (details included in Methods section) are performed in the box geometry and with a rephasing pulse sequence (see Fig. 2a). Briefly, three sub-40-fs pulses with wavevectors **k**_1_, **k**_2_ and **k**_3_ interact nonlinearly with the sample to generate a four-wave mixing signal 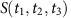 that is detected in the phase-matched direction **k**_S_=**−k**_1_+**k**_2_+**k**_3_. We show the pulse time ordering in Fig. 2b. The four-wave mixing signal is heterodyne detected with a fourth phase-stabilized reference pulse and the resulting interference signal is spectrally resolved using a spectrometer. The signal is recorded as the delay between the first two excitation pulses, *t*_1_, is scanned, while the delay between the second and third pulses, *t*_2_, is held fixed. Fourier transformation of the signal with respect to *t*_1_ generates a 2D rephasing spectrum of the signal 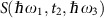, which correlates the excitation energies 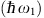 of the system during *t*_1_ with its emission energies 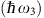 during *t*_3_. For all 2DCS experiments, the fluence of the excitation pulses is kept below ∼4 μJ cm^−2^ (∼10^12^ excitons per cm^−2^) to ensure that the signal is in the *χ*^(3)^ regime and higher-order nonlinearities do not contribute.

**Figure 3:**
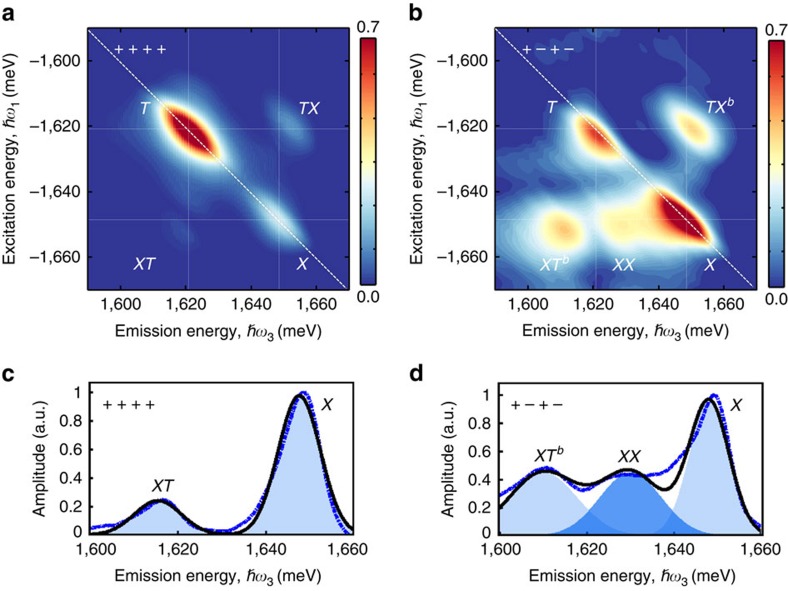
Measured polarization-resolved 2D coherent spectra revealing the biexciton resonances. (**a**) Normalized 2D amplitude spectrum obtained using co-circular polarization of the excitation pulses and detected four-wave mixing signal. The spectrum features two diagonal peaks corresponding to the degenerate excitation and emission of the exciton (*X*) and trion (*T*) resonances and two off-diagonal peaks (*XT* and *TX*) corresponding to their coupling. (**b**) Normalized 2D amplitude spectrum obtained using cross-circular polarization of the first/third fields (*σ*+) and second/signal fields (*σ*−). The additional peak (*XX*) is associated with the neutral bound biexciton, while the shift of the off-diagonal peaks 
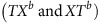
 to lower emission energy is associated with the charged bound biexciton. Slices along the emission energy axis (∼50 μeV resolution) at an excitation energy of 1,648±0.05 meV are shown in (**c**,**d**) for co-circular and cross-circular polarization, respectively. The data are fit with double and triple Gaussian functions (solid lines), respectively, with the individual fits to peaks *XT*^*b*^, *XX* and *X* indicated by the shaded regions.

We first present a 2D amplitude spectrum in Fig. 3a acquired using co-circularly polarized excitation and detection, which is sensitive to excitonic transitions in only one valley. The spectrum features two peaks on the diagonal dashed line at ∼1,648 and ∼1,621 meV corresponding to excitation and emission from the exciton (*X*) and trion (*T*) transitions, respectively. The linewidths of the peaks along the diagonal (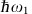
=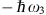) and cross-diagonal (perpendicular to the diagonal) directions reflect the inhomogeneous and homogeneous broadening of the exciton and trion resonances, which have been previously characterized^38^,^42^. The small Stokes shift of the peaks relative to the linear emission spectrum and the moderate inhomogeneous linewidths attest to the quality of the material. The co-circular spectrum also features off-diagonal cross peaks at the excitation energy of the exciton and emission energy of the trion (*XT*) and vice-versa (*TX*), which are indicative of coherent interactions between the exciton and trion^10^,^38^,^43^.

The 2D spectrum obtained using cross-circular polarization shown in Fig. 3b is the main experimental result of this work. An additional peak (*XX*) appears in the spectrum redshifted along the emission energy axis from the exciton, which we attribute to the neutral biexciton state. Within the frame work of perturbation theory of the nonlinear optical response up to *χ*^(3)^, the quantum mechanical pathway responsible for peak *XX* can be understood from the timing sequence in Fig. 2b and the energy level diagram shown in the interaction picture in Fig. 2c as follows: the first pulse (*σ+*) resonantly drives the *g*↔*X* transition to create a coherent superposition (coherence) between the crystal ground state 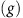 and the exciton state 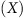 in the *K* valley. After a delay *t*_1_, the second pulse (*σ−*) drives the exciton transition in the *K*′ valley. The Coulomb interaction between the two excitons effectively converts the optical coherences to a dipole-forbidden, non-radiative coherence between excitons in the *K* and *K*′ valleys during *t*_2_. After a delay *t*_2_, the third pulse (*σ+*) excites the exciton-to-biexciton transition corresponding to an optical coherence along *X*↔*XX*, which radiates as the four-wave mixing signal. This quantum pathway evolves during delay *t*_1_ with energy *E*_*X*_ and during delay *t*_3_ with energy *E*_*X*_–Δ_*XX*_, where Δ_*XX*_ is the neutral biexciton binding energy. In the Fourier domain, this contribution appears at the exciton excitation energy *E*_*X*_ and emits redshifted from the exciton peak by Δ_*XX*_, which is consistent with the neutral biexciton peak labelled *XX* with Δ_*XX*_≈20 meV. Because the state *XX* is only observable when the first two excitation pulses have opposite helicity, we conclude that this biexciton consists of two excitons with opposite valley index.

Similar to the formation of the neutral biexciton state *XX*, a charged biexciton state (*XT*^*b*^ or *TX*^*b*^) can be resonantly created. The associated energy diagram is obtained if one exciton state is replaced with one trion state as shown in Fig. 2c (see Supplementary Notes 2–4). Experimentally, this new charged biexciton is identified by the cross peaks *XT*^*b*^ and *TX*^*b*^ redshifted from *XT* and *TX*, respectively, along the emission energy axis by ∼5 meV for cross-circular polarization. This polarization-dependent shift is consistent with the formation of a five-particle charged biexciton arising from a bound state between the exciton and negative trion—an interpretation that is supported by theoretical calculations of the optical response derived using Liouville space pathways^44^, as discussed below. A comparison of slices from the co-circularly and cross-circularly polarized 2D spectra taken along the emission energy axis at the exciton excitation energy, shown in Fig. 3c,d (blue curves), highlights the exciton (*X*), neutral biexciton (*XX*) and charged biexciton (*XT*^*b*^) states. The co-circular (cross-circular) slice is fit with a double (triple) Gaussian function (black curve) to determine the resonance energy of each peak. We emphasize that signatures of the neutral and charged biexcitons observed here are likely masked by the trion transition in linear optical spectra as well as in pump/probe experiments. A comparison between a slice from the 2D spectrum and the projection onto the emission energy axis (analogous to pump/probe spectroscopy) highlights the advantage of 2DCS for isolating the optical response of individual resonances in inhomogeneously broadened materials (see Supplementary Note 1 and Supplementary Fig. 2). We note that the laser bandwidth is broad enough to search for bound states with binding energies as large as 80 meV. If the neutral biexciton binding energy were in the range of 50–80 meV as reported in previous studies of other monolayer TMD materials, we should have observed additional peaks at lower emission energies. The absence of additional lower-energy peaks further supports our assignment of the new peak *XX* between the exciton and trion as the bound neutral biexciton.

## Discussion

**Figure 4:**
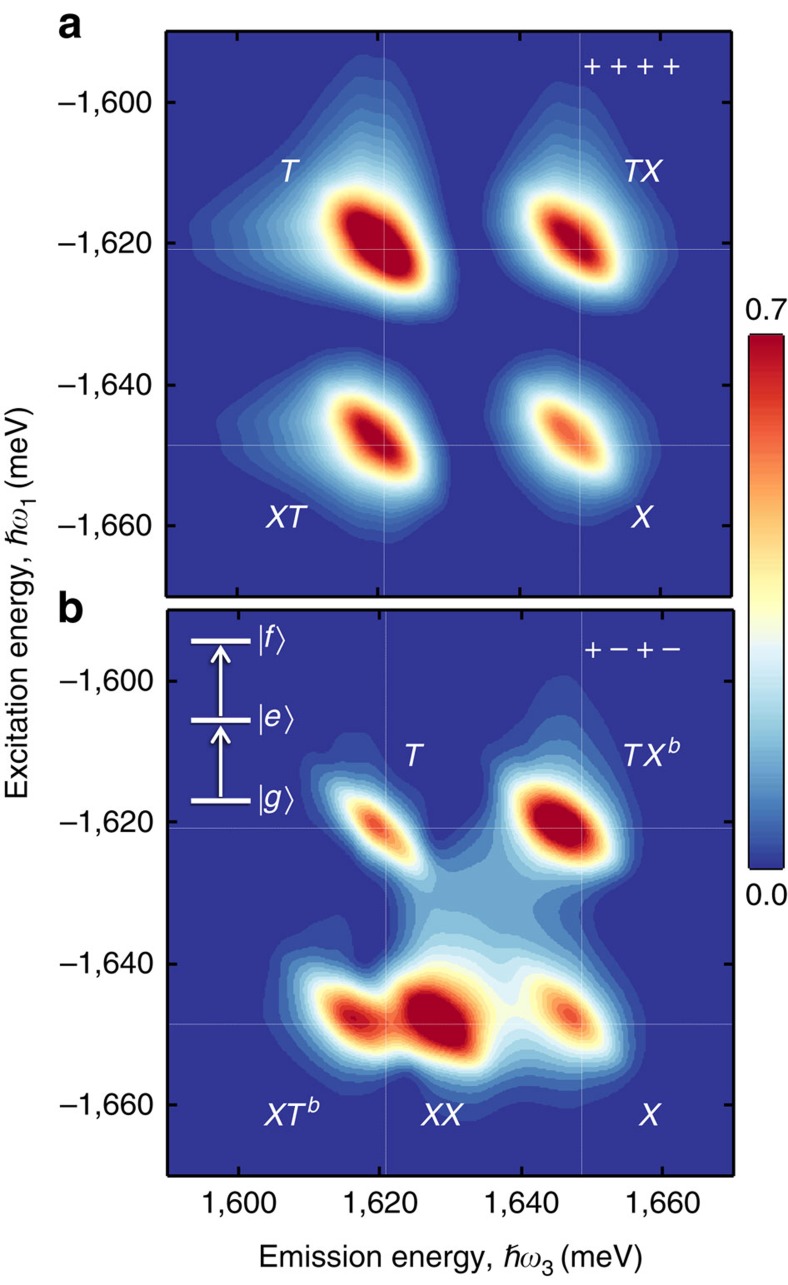
Calculated 2D spectra incorporating the singly- and doubly excited-state manifold. Normalized 2D amplitude spectrum for (**a**) co-circular and (**b**) cross-circular polarization of the excitation pulses and detected signal. In the case of cross-circular polarization, both the singly and doubly excited states indicated by the inset are accessible.

Further insight into the origin of the biexciton states is obtained through calculations of the co- and cross-circularly polarized 2D spectra, which account for exciton–exciton, trion–trion and exciton–trion interactions phenomenologically (see Methods and Supplementary Notes 2–4 for details). The third-order polarization is evaluated for finite pulses in the rephasing time ordering using an energy level scheme consisting of singly and doubly excited manifolds of the exciton and trion states. The valley-specific polarization selection rules are explicitly considered here. A calculated 2D spectrum for co-circular excitation is shown in Fig. 4a. The spectrum features two diagonal and two off-diagonal peaks similar to the measured spectrum shown in Fig. 3a. For this polarization sequence, only the exciton and trion transitions in one valley are relevant. The diagonal population peaks are associated with the singly excited excitons and trions. The off-diagonal coupling peaks appear un-shifted in the excitation and emission energies with respect to exciton and trion resonances and originate from coherent interactions between these two resonances. Due to the co-circular excitation scheme considered here and the valley-specific optical selection rules, many-body effects associated with interaction-induced shifts, or binding between multiple quasiparticles, will not contribute to the signal within the spectral bandwidth since only the singly excited manifolds are accessible.

The simulated spectrum for the case of cross-circular polarized excitation pulses is shown in Fig. 4b—the key features of which are consistent with the measurements (see Supplementary Table 1). Most importantly, the doubly excited-state manifold is now accessible through the *g*→*e*→*f* pathway (inset to Fig. 4b), where *e* and *f* represent one- and two-quasiparticle excitation manifolds (Supplementary Figs 3–5). In the absence of Coulomb interactions between quasiparticles, the transitions *g*→*e* and *e*→*f* are quantum mechanically the same and cannot be distinguished. As a result, the quantum pathways of the singly and doubly excited states completely cancel and the nonlinear signal is zero. Therefore, the fact that a signal is observed for this polarization sequence and for each peak type—diagonal and off-diagonal ones—necessarily implies that many-body effects stemming from exciton–exciton, exciton–trion and trion–trion interactions dominate the nonlinear optical response. The neutral biexciton peak *XX* and the charged biexciton peaks (*XT*^*b*^ and *TX*^*b*^) are present in the calculated cross-polarization spectra with binding energies taken from the experiment in accordance with earlier calculations^28^,^29^,^30^,^31^,^32^.

Interestingly, the weaker trion peak *T* compared to the exciton peak *X* for cross-circular polarization versus the co-circular case in both measured and calculated spectra implies that trion–trion interactions are significantly weaker. Exciton–exciton interactions produce a tightly bound biexciton state, whereas the interaction shift for the trion (Δ_*TT*_) is estimated from a comparison of the exciton and trion relative amplitudes to be an order of magnitude smaller (∼2 meV) likely due to weak localization at potential fluctuations and spatial separation of trions^45^. As a result, the quantum pathways associated with the singly and doubly excited states of the trion destructively interfere and lead to a small amplitude for the trion peak *T*. This interpretation is consistent with the slightly smaller exciton–trion (charged biexciton) interaction shift Δ_*XT*_ observed here compared to theoretical studies reported in the literature thus far, which assume complete delocalization of trions in the 2D plane^28^,^29^,^46^. Note that the spectral position and relative intensity of the trion peak *T* are also influenced by effects such as the electron density in the doped sample and disorder, which are not covered by the phenomenological model applied here.

In summary, we have observed unambiguous spectroscopic features of higher-order correlated states in monolayer MoSe_2_, which we interpret as the neutral and charged biexcitons supported by a phenomenological calculation taking into account valley-contrasting optical selection rules. Furthermore, our interpretation of the biexciton states in TMD is fully consistent with previous 2D spectroscopy studies of biexcitons in other materials systems such as quantum wells, quantum dots and layered GaSe^12^,^47^,^48^. A comparison of the nonlinear optical response under co- and cross-circular polarization conditions reveals the binding energies for the neutral and charged bound biexcitons are ∼20 and ∼5 meV, respectively. These values are consistent with recent theoretical models predicting that the biexciton and trion have a similar electron-hole correlation function^29^. There are a number of ways to reconcile our results with previous studies on biexcitons in other TMDs. One possible explanation is that previous experiments have observed charged biexcitons or excited-state biexcitons^29^. It was not possible to distinguish different types of higher-order bound states based on the one-dimensional spectroscopy methods used in the previous studies. Characterization of the exciton–exciton, exciton–trion and trion–trion binding energies should assist future theoretical studies aiming to better understand many-body interactions in 2D materials and help guide efforts searching for more exotic complexes including even higher-order bound states^49^ and polariton condensates^50^,^51^.

## Methods

### Optical 2DCS

40-fs pulses generated from a mode-locked Ti:Sapphire laser at a repetition rate of 80 MHz are split into a set of four phase-stabilized pulses using a system of nested Michelson interferometers. Three of the pulses are focused to a single ∼30 μm spot on the sample. The coherent interaction of the pulses with the sample generates a four-wave mixing signal that is emitted in the wavevector phase-matched direction. The signal is recorded as the delay *t*_1_ is stepped with interferometric precision. Subsequent Fourier transformation yields a rephasing one-quantum spectrum 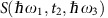. Because the conjugated pulse 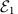 arrives at the sample first, the optical coherences evolve during *t*_1_ with opposite phase as the coherences generated by 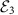 during *t*_3_. As a result, the spectra are plotted with negative excitation energy. We hold *t*_2_=0 fs to obtain maximum signal-to-noise; however, using a value greater than the pulse duration provides similar results at an overall smaller signal amplitude.

### Spin- and valley-selective optical transitions in monolayer MoSe_2_

**Figure 5:**
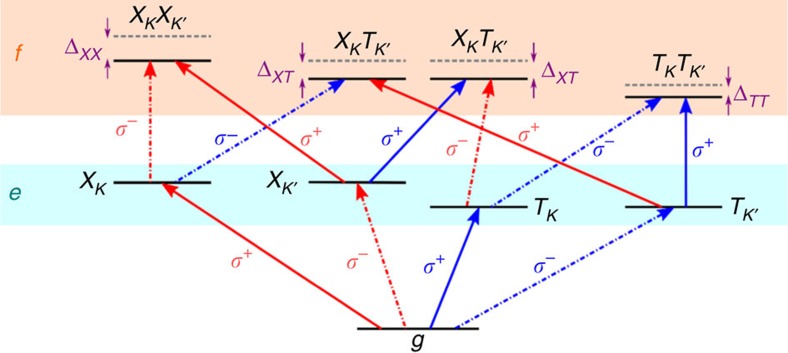
Level scheme used to calculate the optical response for excitation with circularly polarized light. The nine-level energy scheme shows the possible excitation pathways using *σ*+ (solid lines) and *σ*− (dashed-dotted lines) circularly polarized light. Red arrows mark the excitation of uncharged exciton states; blue arrows indicate the excitation of negatively charged exciton states with an additional electron (that is, trions). The level scheme consists of the ground state *g* as well as the singly and doubly excited states in manifolds *e* and *f*. Due to many-body interactions, the doubly excited states are shifted by Δ_*XX*_, Δ_*XT*_ and Δ_*TT*_ with respect to the sum of the individual transitions they are built of (grey, dashed levels).

The nine-level energy scheme used to calculate the third-order nonlinear optical response is depicted in Fig. 5. The possible excitation pathways using *σ*+ and *σ*− circularly polarized light are represented by the solid and dashed-dotted lines, respectively. Red arrows mark the excitation of neutral excitons and blue arrows indicate the excitation of negatively charged excitons (trions). The level scheme consists of a ground state (*g*), a singly excited-state manifold *e*, and a doubly excited-state manifold *f*. The model system considers a common ground state for both the exciton and trion, which represents the background charge carriers in the absence of optically excited electrons and holes. An optical excitation creates an additional electron-hole pair, which can either form a neutral bound exciton or a bound three-particle charged exciton. In the nonlinear *χ*^(3)^ optical response, doubly excited states enter the optical response through inter-valley biexciton (*XX*), trion–trion (*TT*) and exciton–trion (*XT*) states. Many-body interactions are modelled phenomenologically by shifting the energy of these doubly excited states with respect to the sum of the individual transitions from which they are constructed. Further details of the energy level scheme and theoretical calculations are given in the Supplementary Notes 2–4.

### Data availability

The data that support the findings of this study are available from the corresponding authors upon request.

## Additional information

**How to cite this article:** Hao, K. *et al*. Neutral and charged inter-valley biexcitons in monolayer MoSe_2_. *Nat. Commun.* **8**, 15552 doi: 10.1038/ncomms15552 (2017).

**Publisher’s note**: Springer Nature remains neutral with regard to jurisdictional claims in published maps and institutional affiliations.

## Supplementary Material

Supplementary Information Supplementary Figures, Supplementary Tables, Supplementary Notes and Supplementary References
