# Supplementary material for: Neutral and charged inter-valley biexcitons in monolayer MoSe2
Source: Nat Commun. 2017 Jun 28;8:15552. doi: 10.1038/ncomms15552 (PMC5493760; doi:10.1038/ncomms15552)
Supplement: Supplementary Information — Supplementary Figures, Supplementary Tables, Supplementary Notes and Supplementary References [file ncomms15552-s1.pdf]

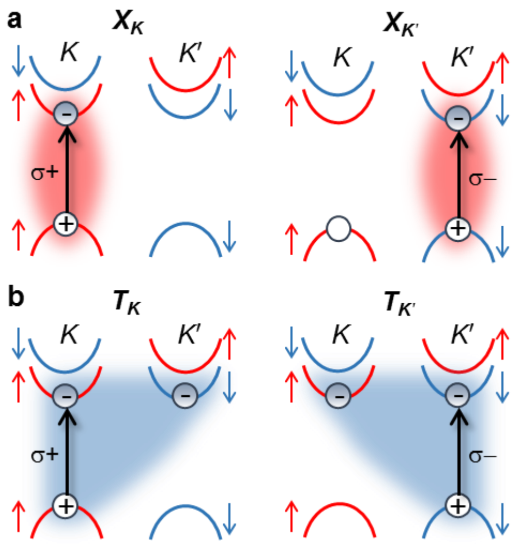

Supplementary Figure 1: Band diagram of (a) excitons and (b) trions in $MoSe_2$ at the *K* and *K′* points.

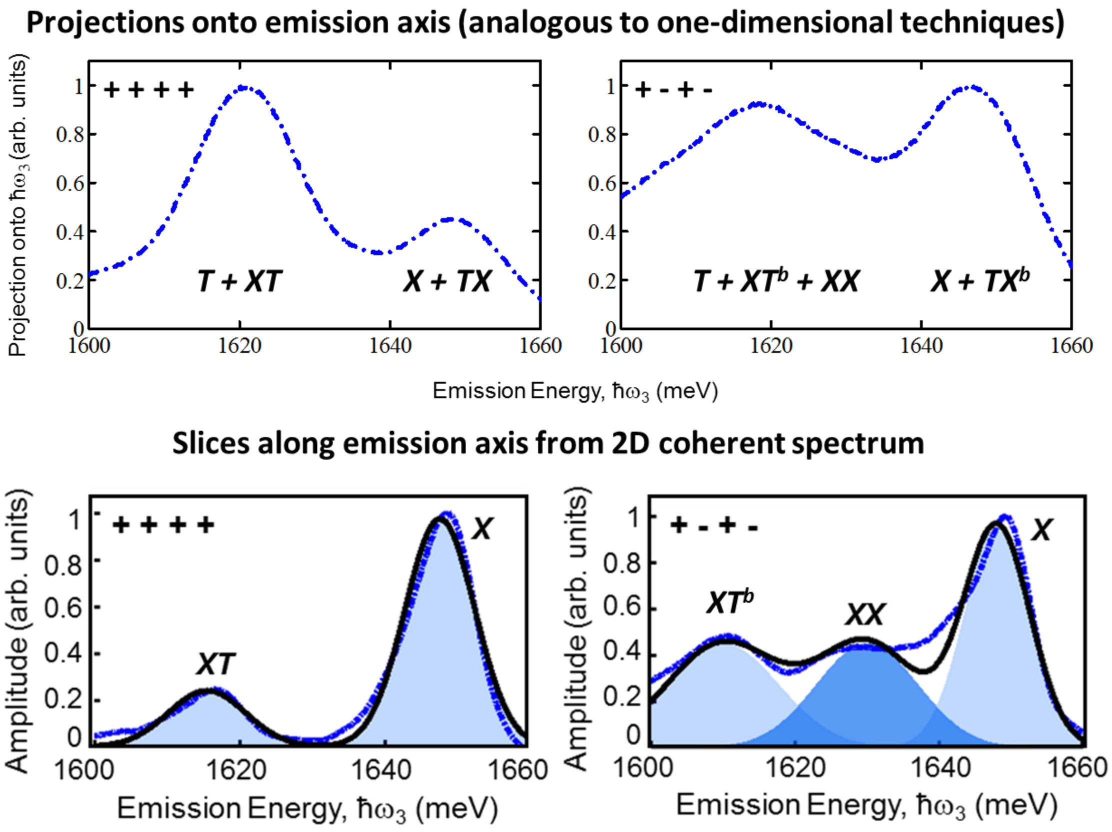

Supplementary Figure 2: Comparison between the projection of the 2D spectrum onto the emission energy axis (top panels and analogous to pump/probe spectra) and a slice from the 2D spectrum along the emission energy axis at the exciton excitation energy (bottom panels).

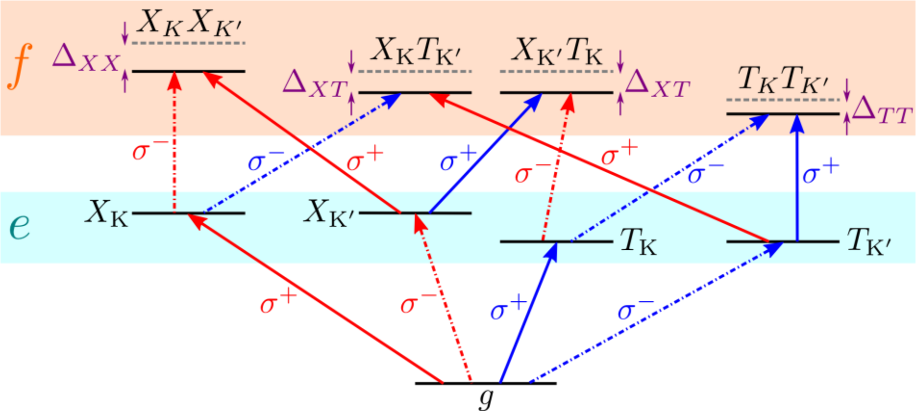

Supplementary Figure 3: Level scheme used to calculate the optical response for excitation with circularly polarized light. The nine-level energy scheme shows the possible excitation pathways using σ^+ (solid lines) and σ^- (dashed-dotted lines) circularly polarized light. Red arrows mark the excitation of uncharged exciton states; blue arrows indicate the excitation of negatively charged exciton states with an additional electron (*i.e.*, trions). The level scheme consists of the ground state g as well as the singly and doubly excited states in manifolds e and f. Due to many-body interactions, the doubly excited states are shifted by Δ_{XX}, Δ_{XT}, and Δ_{TT} with respect to the sum of the individual transitions they are built of (grey, dashed levels).

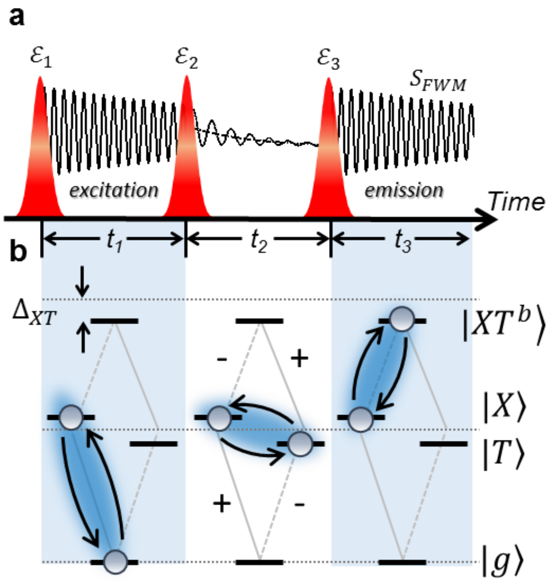

Supplementary Figure 4: The time ordering of the excitation pulses and detected signal and the corresponding quantum pathway for the bound charged exciton peak XT^b are shown in **(a)** and **(b)**, respectively.

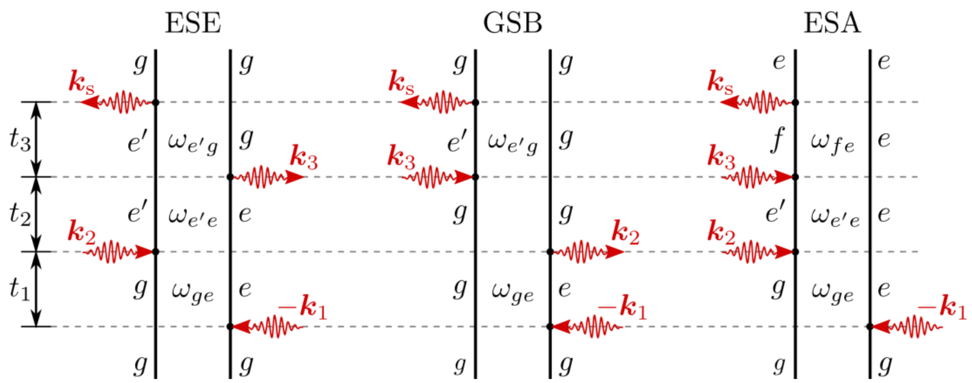

Supplementary Figure 5: Double-sided Feynman diagrams for the rephasing signal generated in the $\boldsymbol{k}_s = -\boldsymbol{k}_1 + \boldsymbol{k}_2 + \boldsymbol{k}_3$ direction. They illustrate the Liouville space pathways (ESE, GSB, and ESA) of the density matrix contributing to the rephasing signal.

	$g \leftrightarrow X$	$X \leftrightarrow XX$	$T \leftrightarrow XT$	$g \leftrightarrow T$	$T \leftrightarrow TT$	$X \leftrightarrow XT$
d_e	1.0			0.8		
E	$E_X = 1648.5$	$E_X - \Delta_{XX}$ $\Delta_{XX} = 20.3$	$E_X - \Delta_{XT}$ $\Delta_{XT} = 5.0$	$E_T = 1620.8$	$E_T - \Delta_{TT}$ $\Delta_{TT} = 2.0$	$E_T - \Delta_{XT}$ $\Delta_{XT} = 5.0$

Supplementary Table 1: Parameter set (dipole moment d_e in e nm and transition energies in meV) used to calculate the 2D spectra given in Fig. 4 in the main text.

Supplementary Note 1: Eliminating Inhomogeneous Broadening using 2DCS

Optical 2DCS has been previously implemented to investigate biexcitons in semiconductor quantum wells and quantum dots. Using 2DCS, the biexciton can be isolated from resonances associated with other quasiparticles in the two-dimensional frequency plane as shown in Fig. 3 of the main text. Supplementary Figure 2 highlights this point. The top two panels depict the projection of the 2D spectrum onto the emission energy axis for co-circular (left) and cross-circular (right) polarization schemes. In this case, several, inhomogeneously broadened contributions to each peak spectrally overlap, which precludes independent analysis of the exciton, trion, and biexciton. This type of projection demonstrates the challenges associated with one-dimensional linear and nonlinear spectroscopy techniques for which inhomogeneity can mask the optical response even in samples with reasonable quality (*i.e.* small-to-moderate Stokes shifts and inhomogeneous broadening). By taking a slice in the 2D spectrum, shown in the bottom panels in Supplementary Figure 1, the effects of inhomogeneous broadening are eliminated and the exciton, neutral biexciton, and charged biexciton can be analyzed independently.

Supplementary Note 2: Spin- and valley-selective optical transitions in monolayer $MoSe_2$

Within the effective mass approximation, a parabolic band structure is used around the optically relevant high-symmetry K and K' points marking the direct band gap of monolayer TMDs. The spin-orbit coupling leads to a pronounced splitting of the spin-up and spin-down valence band. Supplementary Figure 1a shows the lowest allowed optical transitions (so-called A excitons) accessible in monolayer $MoSe_2$ using $\sigma^+$and σ^- circularly polarized light. Note that the highest valence and lowest conduction band spin states are energetically reversely ordered at the K and K' points (cf. Supplementary Figure 1a), such that the optical excitation of the A transition with circularly polarized light is both spin and valley selective. The transition dipole moments are $\mathbf{d}_{eg}^{\mathrm{K}\uparrow} = \frac{d_e}{\sqrt{2}}(1, i)$ and $\mathbf{d}_{eg}^{\mathrm{K}'\downarrow} = \frac{d_e}{\sqrt{2}}(1, -i)$ for the K and K' valleys, respectively. If the sample doping is n-type, linear optical excitations can generate excitons (X) and negatively charged singlet trions (T) in each valley. In the nonlinear $\chi^{(3)}$ regime, doubly excited states enter the optical response such as the intervalley biexciton (XX), trion-trion (TT), and mixed exciton-trion (XT) states. Note that the excited state configurations included in the calculations are the lowest-energy bound states. Other resonances are possible such as B excitons, excited biexcitons, intravalley biexcitons, triplet

trions, etc.[1–6]. However, they are at higher energies than the states considered here and thus are not within the chosen spectral bandwidth.

In Supplementary Figure 3, the polarization-specific optical excitation scheme with the excited state configurations is shown. Exciton-exciton, trion-trion, and exciton-trion forming complexes lead to a Coulomb-induced shift (indicated by the purple arrows in Supplementary Figure 2b) in the energy of the doubly excited states with respect to the sum of the individual transitions they are built of: $E_{XX} = 2E_X - \Delta_{XX}$, $E_{TT} = 2E_T - \Delta_{TT}$, $E_{XT} = E_X + E_T - \Delta_{XT}$.

Note that our model system considers a common ground state for both the exciton and trion. The background charge carrier density of unbound electrons and holes is also present in the ground state[7]. An optical excitation creates an additional electron-hole pair, which can either form a neutral bound exciton state or capture a background carrier to form a bound trion state.

We show in Supplementary Figure 4 one specific pathway corresponding to the bound charged exciton state that appears in the 2D spectrum in Figs. 3b and 4b of the main text as peak XT^b. The excitation pulse sequence and polarization scheme is the same as for the bound neutral biexciton; however, in the present case, the excitation and emission energies of the quantum pathway are E_X and $E_X + E_T - \Delta_{XT}$, respectively.

Supplementary Note 3: Calculation of the rephasing one-quantum signal

In the following, the nonlinear optical response is calculated following Ref. [8]. In a four-wave mixing (FWM) experiment, a sequence of three pulses with defined polarization directions, envelopes $\mathcal{E}_j^{u_j}$, laser frequencies Ω_j, wave vectors $\mathbf{k}_\mathrm{j}$, and variable delay times t_j is applied to the system (cf. Fig. 2b in the main text). Each pulse j is centered at time τ_j:

$$\mathbf{E}(\mathbf{r}, t) = \sum_{j=1}^{3} \sum_{u_j=\pm 1} \mathcal{E}_j^{u_j}(\mathbf{r}, t - \tau_j) \mathrm{e}^{i u_j (\mathbf{k}_j \cdot \mathbf{r} - \Omega_j (t - \tau_j))} \quad (1)$$

The signal depends on the delay times t_i between the pulses (cf. Fig. 1 in the main text):

$$S_{\boldsymbol{k}_\mathrm{s}}^{(3)}(t_3, t_2, t_1) = \int_{-\infty}^{+\infty} \mathrm{d}t \; \mathbf{P}_{\mathbf{k}_\mathrm{s}}(t) \cdot \boldsymbol{\mathcal{E}}_s^*(t - \tau_s) \mathrm{e}^{i\Omega_s (t - \tau_s)} \quad (2)$$

The induced polarization $\mathbf{P}_{\mathbf{k}_\mathrm{s}}(t)$ can be calculated using the third-order response function $R^{(3)}$:

$$P_\alpha^{(3)}(\mathbf{r},t) = \int_0^\infty \int_0^\infty \int_0^\infty \mathrm{d}T_3\, \mathrm{d}T_2\, \mathrm{d}T_1 \sum_{\beta,\gamma,\delta=1}^3 R_{\alpha\beta\gamma\delta}^{(3)}(T_3,T_2,T_1) \times E_\beta(\mathbf{r},t-T_3)E_\gamma(\mathbf{r},t-T_3-T_2)E_\delta(\mathbf{r},t-T_3-T_2-T_1) \quad (3)$$

To extract the rephasing contribution, the heterodyne signal is detected along the phase-matched direction $\mathbf{k}_\mathrm{s} = -\mathbf{k}_1 + \mathbf{k}_2 + \mathbf{k}_3$. The corresponding rephasing 2D spectrum is composed of three contributions given by the double-sided Feynman diagrams in Supplementary Figure 5. The three quantum pathways are excited-state emission (ESE), ground-state bleaching (GSB) and excited-state absorption (ESA). The rephasing one-quantum signal in the frequency domain is calculated by Fourier transforming the third-order heterodyne-detected signal with respect to the time intervals t_1 and t_3 between the pulses. The three contributions have the form[8]

$$S_{\mathrm{ESE}}^{(3)}(\omega_3,t_2,\omega_1) = \frac{(2\pi)^4}{\hbar^3}\sum_{e,e'} \left(\mathbf{d}_{e'g}^* \cdot \boldsymbol{\mathcal{E}}_\mathrm{s}^*(\omega_{e'g}-\Omega_s)\right)\left(\mathbf{d}_{e'g}\cdot \boldsymbol{\mathcal{E}}_2(\omega_{e'g}-\Omega_2)\mathrm{e}^{-i\xi_{e'e}t_2}\right) \times \frac{\mathbf{d}_{eg}\cdot\boldsymbol{\mathcal{E}}_3(\omega_{eg}-\Omega_3)}{\omega_3-\xi_{e'g}}\frac{\mathbf{d}_{eg}^*\cdot\boldsymbol{\mathcal{E}}_1^*(\omega_{eg}-\Omega_1)}{\omega_1-\xi_{ge}} \quad (4)$$

$$S_{\mathrm{GSB}}^{(3)}(\omega_3,t_2,\omega_1) = \frac{(2\pi)^4}{\hbar^3}\sum_{e,e'} \left(\mathbf{d}_{e'g}^* \cdot \boldsymbol{\mathcal{E}}_\mathrm{s}^*(\omega_{e'g}-\Omega_s)\right)\left(\mathbf{d}_{eg}\cdot \boldsymbol{\mathcal{E}}_2(\omega_{eg}-\Omega_2)\right) \times \frac{\mathbf{d}_{e'g}\cdot\boldsymbol{\mathcal{E}}_3(\omega_{e'g}-\Omega_3)}{\omega_3-\xi_{e'g}}\frac{\mathbf{d}_{eg}^*\cdot\boldsymbol{\mathcal{E}}_1^*(\omega_{eg}-\Omega_1)}{\omega_1-\xi_{ge}} \quad (5)$$

$$S_{\mathrm{ESA}}^{(3)}(\omega_3,t_2,\omega_1) = -\frac{(2\pi)^4}{\hbar^3}\sum_{e,e',f} \left(\mathbf{d}_{fe}^* \cdot \boldsymbol{\mathcal{E}}_\mathrm{s}^*(\omega_{fe}-\Omega_s)\right)\left(\mathbf{d}_{e'g}\cdot \boldsymbol{\mathcal{E}}_2(\omega_{e'g}-\Omega_2)\mathrm{e}^{-i\xi_{e'e}t_2}\right) \times \frac{\mathbf{d}_{fe'}\cdot\boldsymbol{\mathcal{E}}_3(\omega_{fe'}-\Omega_3)}{\omega_3-\xi_{fe}}\frac{\mathbf{d}_{eg}^*\cdot\boldsymbol{\mathcal{E}}_1^*(\omega_{eg}-\Omega_1)}{\omega_1-\xi_{ge}} \quad (6)$$

with $\xi_{ab} \equiv \omega_{ab} - i\gamma_{ab}$. $\mathbf{d}_{ab}$ denotes the dipole moment, ω_{ab} the resonance energy and γ_{ab} the homogeneous broadening of the $b \to a$ transition, $\mathcal{E}_i$ is the envelope of the i-th pulse. e and e' label the singly excited state manifold and f the doubly excited state manifold. Note that the ESA pathway is the only contribution incorporating the doubly-excited state manifold. The total (measured) photon-echo signal is the sum of the three contributions:

$$S_{\mathrm{tot}}^{(3)}(\omega_3,t_2,\omega_1) = S_{\mathrm{ESE}}^{(3)}(\omega_3,t_2,\omega_1) + S_{\mathrm{GSB}}^{(3)}(\omega_3,t_2,\omega_1) + S_{\mathrm{ESA}}^{(3)}(\omega_3,t_2,\omega_1) \quad (7)$$

Note that cross peaks in the spectra indicate coherent couplings between the excited states, since many-body interactions break the symmetry between the $g \leftrightarrow e$ and $e \leftrightarrow f$ transitions. As a

consequence, the Liouville space pathways involving singly excited states (lower transitions) are not canceled by the pathway including the doubly excited states (upper transitions).

The possible optical transitions with their dipole strengths and energies are listed in Supplementary Table 1. The exciton states X_K and $X_{K'}$ are denoted as X, the trion states T_K and $T_{K'}$ as T, and the two mixed exciton-trion states $X_K T_{K'}$ and $X_{K'} T_K$ as XT, since they are of the same type (but can be selected using circularly polarized light). Note that in Figs. 3 and 4 of the main text, the two exciton-trion cross-peaks below and above the diagonal are labeled XT and TX, respectively, and are marked by a superscript (XT^b and TX^b) when they are interaction shifted.

Inhomogeneous broadening is included by averaging the signal functions for normally distributed values of the system resonances. We assume a perfect correlation of the transition energies during t_1 and t_3: the changes in transition energy during t_1 are directly correlated to the changes in transition energy during t_3. This is a reasonable assumption, since the (fixed) delay time t_2 between the second and third pulse is vanishing and therefore no spectral diffusion processes will take place between t_1 and t_3. The inhomogeneous broadening is set to $\gamma_{\mathrm{inh}} =$ 4 meV. It leads to elongated peaks along the diagonal ($\hbar\omega_1 = -\hbar\omega_3$). The broadening of the cross-diagonal is determined by the homogeneous linewidth, which was set to $\gamma_{\mathrm{hom}} = 2.0$ meV for all transitions.

In order to consider laser bandwidth effects, we approximate the excitation laser spectrum by a Gaussian-shaped spectral distribution with the half width at half maximum $\gamma_{\mathrm{laser}} = 30$ meV and the central wavelength of the pulse $\lambda_{\mathrm{c}} = 765$ nm (coinciding with the trion resonance). All parameters were chosen in agreement with the experiment.

Supplementary Note 4: Discussion of the calculated spectra

The calculated rephasing one-quantum signals for two different polarization combinations are shown in Fig. 4 in the main text. Fig. 4a displays the calculated 2D spectrum for co-circular excitation. The spectrum exhibits two diagonal peaks at $(E_X, -E_X)$ and $(E_T, -E_T)$ as well as two off-diagonal peaks at $(E_T, -E_X)$ and $(E_X, -E_T)$. The optical selection rules (cf. Supplementary Figure 2a) ensure that only one valley is addressed using a co-circular excitation scheme within the considered spectral bandwidth. Therefore, the ESA quantum pathway involving bound biexciton states composed of one (charged) exciton per valley is not excited and the doubly-excited state manifold does not enter the nonlinear optical response. As a result, higher-order bound many-

body states will not contribute to the signal. The diagonal population peaks arise from ESE and GSB nonlinearities associated with the singly-excited state manifold. The lower (upper) off-diagonal cross peak combines the exciton (trion) excitation energy with the trion (exciton) emission energy. These two cross-diagonal peaks would cancel in the absence of many-body interactions.

The situation changes in the case of cross-circular excitation, where the doubly-excited state manifold is accessible through the $g \rightarrow e \rightarrow f$ (ESA) pathway and additional spectral features appear in the simulated spectrum shown in Fig. 4b (main text), which are consistent with the measurements shown in Fig. 3b (main text). These additional peaks indicate the formation of intervalley doubly-excited states composed of two quasi-particles coupled via Coulomb interaction. This time, GSB pathways do not contribute, since they require the same helicity of the first and second excitation pulses (cf. Supplementary Figure 5). Since the ESA pathways enter with opposite sign as the ESE pathways (cf. Supplementary Equations 1 and 5), the two pathways would completely cancel in the absence of interaction-induced energy shifts of the doubly-excited states f. However, the $e \rightarrow f$ transition energies are reduced compared to the corresponding $g \rightarrow e$ excitation energies due to the binding energies of the exciton-exciton (Δ_{XX}), trion-trion (Δ_{TT}), and exciton-trion (Δ_{XT}) complexes. The ESA pathway associated with the biexciton composed of one exciton per valley therefore gives rise to the biexciton peak XX which is spectrally shifted from the exciton peak X by $\Delta_{XX} = 20.3$ meV (cf. Supplementary Table 1) along the emission energy axis. This value is nearly identical to the biexciton binding energy predicted from microscopic calculations[2,3]. The off-diagonal coupling peaks XT^b and TX^b are red-shifted by a few meV along the emission energy axis compared to the spectrum for co-circular excitation. This effect is directly connected to the ESA contributions involving doubly excited charged five-particle bound states $X_{K'}T_K$ and $X_K T_{K'}$ composed of an exciton in one and a trion in the other valley. The associated exciton-trion binding energy $\Delta_{XT} = 5$ meV is considerably smaller than the stronger bound biexciton state XX, which is in line with previous experimental and theoretical studies[3,9]. Since Δ_{XT} is in the same order of magnitude as the homogeneous and inhomogeneous broadening, the un-shifted cross-diagonal XT and TX peaks stemming from the singly-excited state contribution (ESE pathways) spectrally overlap and interfere with the doubly-excited exciton-trion peaks from the ESA contribution. A similar interference between ESE and ESA pathways for the trion peak T occurs with an estimated trion-trion binding energy of $\Delta_{TT} = 2$ meV. Due to this

small binding energy, the $g \rightarrow T$ transition energy is not well separated from the $T \rightarrow TT$ transition and therefore they partly cancel each other out, which leads to a smaller overall amplitude of the superposed trion T peak compared to the exciton peak X. Note that this phenomenological model only provides an estimate of the trion-trion and exciton-trion binding energies, since effects such as doping concentrations, sample quality, excitation power, degree of circular polarization, higher-order effects beyond $\chi^{(3)}$, etc. may have appreciable impact on the spectral signatures and the bound quasi-particle states.

Supplementary References

1. Zhang, C., Wang, H., Chan, W., Manolatou, C. & Rana, F. Absorption of light by excitons and trions in monolayers of metal dichalcogenide MoS_2: Experiments and theory. *Phys. Rev. B* **89,** 205436 (2014).
2. Zhang, D. K., Kidd, D. W. & Varga, K. Excited Biexcitons in Transition Metal Dichalcogenides. *Nano Lett.* **15,** 7002–7005 (2015).
3. Kylänpää, I. & Komsa, H.-P. Binding energies of exciton complexes in transition metal dichalcogenide monolayers and effect of dielectric environment. *Phys. Rev. B* **92,** 205418 (2015).
4. Sie, E. J., Frenzel, A. J., Lee, Y.-H., Kong, J. & Gedik, N. Intervalley biexcitons and many-body effects in monolayer MoS_2. *Phys. Rev. B* **92,** 125417 (2015).
5. Sergeev, R. A. & Suris, R. A. Singlet and triplet states of X+ and X- trions in two-dimensional quantum wells. *Nanotechnology* **12,** 597–601 (2001).
6. Sanvitto, D. *et al.* Origin of the oscillator strength of the triplet state of a trion in a magnetic field. *Phys. Rev. Lett.* **89,** 246805 (2002).
7. Esser, A., Zimmerman, R. & Runge, E. Theory of trion spectra in semiconductor nanostructures. *Phys. Stat. Sol. B* **227,** 317–330 (2001).
8. Abramavicius, D., Palmieri, B., Voronine, D. V., Sanda, F. & Mukamel, S. Coherent multidimensional optical spectroscopy of excitons in molecular aggregates; quasiparticle versus supermolecule perspectives. *Chem. Rev.* **109,** 2350–2408 (2009).
9. Singh, A. *et al.* Coherent Electronic Coupling in Atomically Thin $MoSe_2$. *Phys. Rev. Lett.* **112,** 216804 (2014).
